# Is Genetic Engineering a Route to Enhance Microalgae-Mediated Bioremediation of Heavy Metal-Containing Effluents?

**DOI:** 10.3390/molecules27051473

**Published:** 2022-02-22

**Authors:** Saeed Ranjbar, Francisco Xavier Malcata

**Affiliations:** 1 LEPABE—Laboratory for Process Engineering, Environment, Biotechnology and Energy, Rua Dr. Roberto Frias, s/n, 4200-465 Porto, Portugal; up202000001@edu.fe.up.pt; 2 Department of Chemical Engineering, University of Porto, Rua Dr. Roberto Frias, s/n, 4200-465 Porto, Portugal

**Keywords:** heavy metals, bioremediation, microalgae, genetic engineering, phycoremediation

## Abstract

Contamination of the biosphere by heavy metals has been rising, due to accelerated anthropogenic activities, and is nowadays, a matter of serious global concern. Removal of such inorganic pollutants from aquatic environments via biological processes has earned great popularity, for its cost-effectiveness and high efficiency, compared to conventional physicochemical methods. Among candidate organisms, microalgae offer several competitive advantages; phycoremediation has even been claimed as the next generation of wastewater treatment technologies. Furthermore, integration of microalgae-mediated wastewater treatment and bioenergy production adds favorably to the economic feasibility of the former process—with energy security coming along with environmental sustainability. However, poor biomass productivity under abiotic stress conditions has hindered the large-scale deployment of microalgae. Recent advances encompassing molecular tools for genome editing, together with the advent of multiomics technologies and computational approaches, have permitted the design of tailor-made microalgal cell factories, which encompass multiple beneficial traits, while circumventing those associated with the bioaccumulation of unfavorable chemicals. Previous studies unfolded several routes through which genetic engineering-mediated improvements appear feasible (encompassing sequestration/uptake capacity and specificity for heavy metals); they can be categorized as metal transportation, chelation, or biotransformation, with regulation of metal- and oxidative stress response, as well as cell surface engineering playing a crucial role therein. This review covers the state-of-the-art metal stress mitigation mechanisms prevalent in microalgae, and discusses putative and tested metabolic engineering approaches, aimed at further improvement of those biological processes. Finally, current research gaps and future prospects arising from use of transgenic microalgae for heavy metal phycoremediation are reviewed.

## 1. Introduction

Heavy metals (HMs) are an integral constituent of the biosphere; they are naturally recycled in the environment through various biotic and abiotic processes, as part of biogeochemical cycles [1,2]. However, the dramatic rise in urbanization and industrialization has led to the release of alarmingly toxic levels of HMs, along with many other organic and inorganic pollutants in the environment. Aside from geochemical processes beyond one’s control, as is the case of erosion, atmospheric deposition, infiltration, thermal spring activity, and volcanic eruptions, HMs are increasingly penetrating aquatic systems, as a consequence of a wide range of anthropogenic activities, e.g., discharges of untreated effluents from mining, spontaneous leaching from intensive agriculture, petroleum refining, improved performance of petroleum-based fuels, refuse burning, electroplating, printing, power generation, and other such activities carried out by (fine) chemical and metallurgical industries in the manufacture of microelectronic devices, paints, plastics, batteries, cosmetics, and medical equipment [3,4].

**Figure 1:**
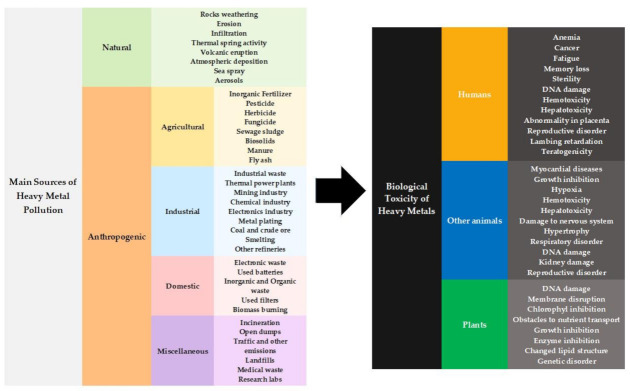
Main sources of heavy metal pollution and biological toxicity thereof.

Generally speaking, 67 out of 118 chemical elements with atomic numbers above 20 and density greater than 5 g.cm^−3^ are considered as HMs [5]; arsenic (As), cadmium (Cd), chromium (Cr), lead (Pb), mercury (Hg), copper (Cu), zinc (Zn), and nickel (Ni) rank among those most repeatedly found at toxic concentrations in water, soil, sediments, and even living organisms in the latest decades [6]. A few HMs are stable as such, and all of them are obviously non-biodegradable, so they will persist as environmental contaminants, with their toxicity posing major health concerns. Most of the harmful effects of HMs upon various vital organs in humans, and inhibition of such basic physiological processes as photosynthesis, mineral nutrition, and water relation, have been well-documented in plants and other organisms [7,8]. Because of interspecies differences in metal stoichiometry, the intracellular concentration of HMs may range from nano- to femtomolar levels [5,9]. Although trace quantities of some HMs are essential for many metabolic processes—especially as cofactors of enzymes. HMs tend to bind to functional groups of biomolecules and destroy their functionality when at high concentration, thus, adversely affecting basic metabolic processes. Examples include disruption of cell membrane permeability, formation of nonfunctional protein–metal adducts, alterations of redox state of cells, generation of toxic free radicals and reactive oxygen species, and direct damage to DNA [10,11,12]—see Figure 1. Therefore, remediation of HM-contaminated terrestrial and aquatic ecosystems is of the utmost importance, in attempts to restore the altered ecological balance of our planet [4,13].

Conventional methods to remove HMs from effluents are chemical precipitation, solvent extraction, ion exchange, evaporation, adsorption, nanofiltration, ultrafiltration, reverse osmosis, and electrochemical treatments; unfortunately, they often prove inefficient in terms of energy input, environmental footprint, capital investment, and operational costs. As a consequence, huge amounts of non-treated domestic and industrial waste end up in the natural environment [2,4,14]. Bioremediation comes as a tool to avoid such dumping (bearing several well-documented advantages [15,16,17,18]); it is defined at large as the application of biological organisms and their components to degrade, transform, sequester, mobilize, or contain environmental contaminants present in soil, water, or air [19]. Various species of plants, bacteria, fungi, yeasts, and microalgae, as well as dead biomass derived therefrom, have shown a great potential for bioremediation of HM-derived pollution in aqueous media—through metal binding and uptake [20,21,22]. Among these, phycoremediation (i.e., use of microalgae for mitigation of organic and inorganic contamination) offers several advantages; hence, it has accordingly undergone intensive investigation for the large-scale remediation of industrial and domestic effluents, as well as HM-contaminated sites and water bodies [12,23].

Microalgae are photoautotrophic eukaryotic microorganisms, which account for over 40% of the global primary production, for lying at the bottom of the aquatic food chain; this is also true for most of the biologically sequestered trace metals in aquatic environments [24]. They are ubiquitously found in nature, as they are well-adapted to live/survive in a wide range of aquatic habitats, from sea and fresh water, through domestic and industrial effluents, to salt marshes and constructed wetlands [25]. Their unique metabolic plasticity, inherent capacity to grow on nonarable lands and in wastewater, using just solar light as the source of energy and atmospheric CO_2_ as the carbon source, and relatively high rate of cell division and growth account for such widespread occurrence; the exceptional retention capacity of HMs also contributes to make them the ideal platform to develop the next-generation technologies for wastewater treatment. Microalgae cells can accumulate HMs up to 10% of their biomass, owing to their large surface-to-volume ratio, coupled with their efficient metal binding, uptake, metabolization, and storage mechanisms [12,22]. The toxicity dosage of HMs is quite variable though, even among members of the same taxonomic group; for instance, some diatoms can tolerate 1.5–10 µM of dissolved Cu, whereas certain species of Chlorophyta still survive in 15 µM [26,27]. Utilization of microalgae for bioremediation of wastewater brings about, in addition, the opportunity to produce a wide range of bioactive products, including proteins, pigments, and vitamins, for eventual use as feed and food additives, and as cosmetic ingredients; lipids and carbohydrates, for eventual production of biofuels and nutritional supplements; a long list of other value-added byproducts, such as biofertilizers and biochar.

Despite their outstanding potential, current technical and economic constraints—associated to the underlying upstream and downstream processes—have hampered large-scale use of microalgae in HM bioremediation [28]. Even though integration of microalgae-mediated wastewater treatment with energy production appears logical and inevitable, the strains most commonly employed, essentially retrieved from nature in their native state, lack the robustness required by sustainable, large-scale scenarios bearing a commercial interest. Recent advances in genetic and protein engineering, complemented by an essentially unanimous orientation toward a holistic approach to the engineering of biological systems, at the expense of bioinformatic and omics tools, may soon allow for the tailor-made design of microbial cell factories for a number of end- or start-products, while lowering the risks and concerns over the putative adverse effects associated to use of genetically modified organisms (GMOs). Further to knowledge on the most obvious routes to enhance energy load (stemming from hydrocarbons) and improving growth rate and photosynthetic ability in microalgae cells, effective engineering of (sustainable) cell factories, for efficient HM bioremediation, will call for work on specific genes and traits identified as relevant.

The present review accordingly summarizes recent advances in the genetic engineering of microalgae, aimed at improving their HM removal capacity and specificity. Furthermore, candidate genes and potential routes susceptible of manipulation are discussed, in attempts to create robust strains of microalgae, able to convey sustainable removal of HMs from wastewater.

## 2. Cellular Mechanisms of HM Bioremediation in Microalgae

Heavy metals adversely affect the physiological health of, and may even cause severe toxicity to microalgae cells, owing to attenuation of the bioactivity of proteins, lipids, nucleic acids, pigments, and other molecules, or the generation of excessive reactive oxygen species (ROS); more specifically, they act by impairing photosynthetic machinery, inhibiting enzyme activities, and/or ceasing cell division [29], or else by inhibiting the normal function of thylakoid membrane and chlorophyll biosynthesis, acidifying cytoplasm, or damaging the cell membrane [30,31,32].

**Figure 2:**
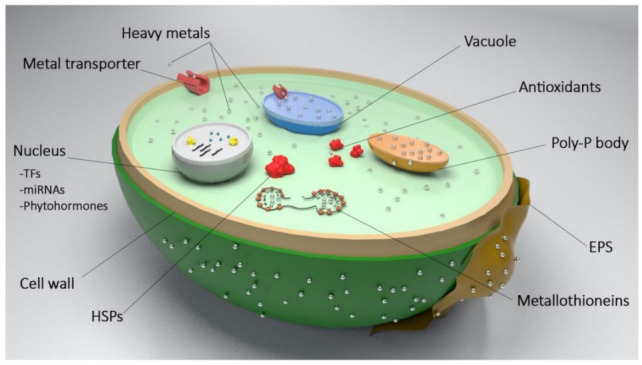
Heavy metal bioremediation mechanisms adapted by microalgae.

Similar to other life forms, microalgae have developed, through evolution, several intracellular and extracellular adaptive mechanisms for the mitigation of HM toxicity—see Figure 2. For instance, the physicochemical properties of the microalgal cell wall and extracellular polymeric substances (EPS) allow the binding of HM ions to functional groups on their surface, in a process generally known as biosorption [33]. As the interface between intracellular compartment and external environment, the constitutive macromolecules of the cell wall possess various negatively charged functional groups, e.g., amino, hydroxyl, carboxyl, sulfhydryl, sulfate, phosphate, carbonyl, amide, imidazole, thioether, and phenol; said moieties can bind to ions from the surrounding medium, in the absence of steric or conformational barriers [34,35,36,37]. The molecular mechanisms behind the biosorption of HMs onto the cell wall and EPS include ion exchange, chelation and complexation, hydroxide condensation, covalent binding, redox interaction, biomineralization, and precipitation of insoluble metal complexes, through electrostatic, van der Waals, or hydrophobic interactions of positively charged HM cations with negatively charged groups present on the cell surface [1,20,38]. The adsorption capacity of the microalgal cell wall is a metabolism-independent process; hence, it is primarily affected by such environmental factors as pH, temperature, contact time, and concentration of HM and competing ions [4,39]. Given the numerous reports on fluidity and evolution of cell wall components (e.g., fatty acids) in response to external stimuli, a yet unknown metabolic background to this phenomenon seems to exist. On the other hand, biosorption of HMs onto EPS is regulated by the cell itself via changes in the properties of such biopolymers, as required by the nature of metabolic stress, i.e., metal toxicity in the situation under scrutiny [40,41]. Both the cell wall and EPS provide, indeed, an extracellular protective layer to the cell that prevents the harmful effects of HMs, if transported into the intracellular compartment; in this fashion, cellular integrity is maintained. The distinct physiology of existing species of microalgae then accounts for the differences found in composition and structure of such outer structures, which, in turn, drive their species- and even strain-dependent HM-biosorption capacities [1,20]. Secretion of metal-chelating proteins and specific organic acids, and subsequent endocytosis of the organometallic complexes formed, is another mechanism for extracellular HM-bioremediation, reported in microalgae cells [42].

Since HMs are hydrophilic in nature, a requirement exists for certain carrier molecules that facilitate their transport into the cells. Once in the cytoplasm, HM toxicity is overcome by resorting to unique metabolic mechanisms—some of which have been well-documented. Several metal efflux pumps do regulate the algal membrane permeability, by actively transporting HMs into and out of the cell [43,44,45]; the net metal flux is accordingly reduced, and may even affect the chemical speciation of the HMs, due to expulsion of trace metal complexes [46].

Another strategy followed by microalgae is increased expression of metal-binding amino acids, peptides, and proteins, such as metallothioneins (MTs), phytochelatins (PCs), glutathione (GSH), proline, histidine, and glutamate [47]. These organometallic complexes are typically transported in, but partitioned into vacuoles—so as to neutralize the otherwise toxic effects of HMs in the cytoplasm [5]. In the acidic environment of vacuoles, HMs are released from their organic carrier; while the latter may be transported back to the cytosol, HMs are most likely stabilized and chelated by sulfides or organic acids in said vacuoles [10,48,49]. Depending on the prevailing environmental conditions, microalgae may attain a high polyphosphate content, suitable for binding divalent HM cations, and drive them into vacuoles for further sequestration. In addition to vacuoles, excess intracellular HM loads can be transported for eventual sequestration in such other organelles as mitochondria and chloroplasts [45], meaning that the expression level of metal transporters in the membrane of those organelles will play an important role in determining HM-removal specificity, capacity, and rate by microalgae strains.

As happens with other cell types, microalgae respond to HM-generated oxidative stress by controlling the cell redox state and overexpressing heat shock proteins (HSPs) [50,51]. MicroRNAs (miRNAs) also serve as key components of the gene regulatory network involved in cellular HM mitigation. They contribute to post-transcriptional cleavage and translational inhibition of target mRNAs, or methylation of target DNAs to regulate a particular response, aimed at maintaining cellular homeostasis, by triggering complexation of excess HMs, defense against oxidative stress, and signal transduction for biological control purposes [52]. Despite the scarce information available on the subject, metal-responsive transcription factors (TFs) appear to activate multiple genes responsible for HM uptake, transport, and detoxification, thus, establishing a global resistance network against HM toxicity in the cell. Therefore, identification and characterization of the set of TFs able to regulate HM stress will be of the utmost importance, in attempts to develop transgenic microalgae with improved bioremediation potential [29,53].

Sexual reproduction, expression of metal-modifying enzymes, and phenotypic plasticity are alternative mechanisms entertained by microalgae as survival tools against HM toxicity [49]. For instance, transcriptomic analysis of *Chlorella vulgaris*, following exposure to Cu cations, unfolded a marked increase in intracellular carotenoids and proline contents, and in activity of such antioxidative enzymes as catalase, peroxidase, polyphenol oxidase, and superoxide dismutase. Photosystem II (PSII) and CO_2_ assimilation are apparently inhibited in microalgae, as a response to metal stress; a relative reduction in growth rate and cell density has been reported for various species of microalgae upon exposure to high concentrations of Cu. A severe drop in protein levels, in parallel to an enhanced rate of carbohydrate biosynthesis have been demonstrated in microalgae cells in the presence of HMs [54,55]. The aforementioned coordinated response of microalgae cells to metal toxicity is a result of crosstalk among different molecular networks, urged by the need for keeping cellular hemostasis. Similar to other cellular functions, the key role played by such signaling molecules as phytohormones, Ca itself, and kinases is worthy of further research [29,56].

## 3. Genetic and Metabolic Engineering Tools for Microalgal Strain Improvement

Most microalgal molecular tools available so far have been developed for *Chlamydomonas reinhardtii,* as model microalga. However, the genomic DNA of several other species of microalgae has meanwhile been sequenced; hence, there is an unprecedented opportunity to manipulate microalgae cells in attempts to improve the efficiency of photosynthesis, carbon assimilation, and production of specific bioproducts—as well as bioremediation of HMs [57]. In fact, recent advances in genome sequencing and reconstruction, gene targeting and transformation, bioinformatics, and multiomics technologies have significantly facilitated the design and manipulation of metabolic pathways in microalgae [57]. Under a holistic systems approach, current gene technology permits relatively rapid and straightforward reconstruction of microalgae genomes, by resorting to state-of-the-art synthetic biology tools, meant to overcome putative biophysicochemical inadequacies of the cell, when responding to a certain environmental condition, or in expressing a desired phenotype [58].

The molecular toolkits developed for microalgal bioengineering include various gene editing technologies of zinc-finger nucleases (ZFNs), transcription activator-like endonucleases (TALENs), clustered regulatory interspaced short palindromic repeats (CRISPR)/Cas systems, Cre/*loxP* recombination systems, RNA interference (RNAi), and modular cloning systems, as well as a long list of promoters, vectors, reporter genes, and regulatory elements [59]. High-capacity gene stacking toolkits, e.g., Golden Gate Modular Cloning (MoClo), have also been established for *Nannochloropsis* and *Chlamydomonas* strains, thus, providing a library of molecular building blocks for the genetic engineering of microalgae cells [60,61]. The Characterization and design of microalgae-specific constitutive, inducible, and synthetic promoters have also improved the flexibility and efficiency of the bioengineering process [62,63]. These molecular tools can be employed to target any desired DNA sequence for the generation of knockout, knockdown, or insertion mutants. Modulation of the simultaneous expression of multiple genes, and creation of markerless and transgene-free knockout mutants are other novel capabilities endowed by recent technological advancements in microalgal molecular cloning [64,65].

Through rational design, directed evolution, *de novo* design, and computational approaches, protein engineering will allow optimization of the protein function, or incorporation of novel functional treats; for instance, two or more domains from distinct proteins can be combined in a fusion protein, expected to bear multiple functions, improved catalytic properties, and higher stability [66]. Furthermore, machine learning is accelerating this process through the mathematical prediction of sequence-to-function correlations, in a data-driven manner—via learning from the properties of variants already characterized [67].

On the other hand, the information necessary to find the right genes for manipulation in biological systems is now widely provided by multiomics approaches. Genomics, transcriptomics, proteomics, metabolomics, and interactomics data, mined from many microalgae strains and grown under various environmental conditions, convey a reliable overview of the patterns of genetic adaptation, molecular evolution, and dynamic changes in microalgal metabolism [68,69,70,71,72]. Several online platforms, including ChlamyCys, Greenhouse, Diatom EST database, Alga-PrAs, Cyan-Omics, Phytozome, Algal Functional Annotation Tool, and Kyoto Encyclopedia of Genes and Genomes (KEGG), already offer freely available omics data, produced from thousands of chromatography, mass spectrometry, nuclear magnetic resonance, and other omics-based experiments [73,74,75].

Genome-scale reconstruction, based on flux balance analysis (FBA), and the mathematical modeling of microalgal metabolic networks is a novel holistic approach for the tailor-made design of cell factories aimed at maximum efficiency; it is already available for some species of microalgae and has met with success in efforts to maximize hydrogen productivity and light usage by *C. reinhardtii* [76,77]. Novel sequencing techniques based on microarray technology and Illumina-based, *de novo* RNA sequencing—which require no prior knowledge of gene sequences in the organism under scrutiny—offer a potent means to the profile gene expression of microbial communities upon exposure to HMs [78,79,80,81]. Meta-omics resorts, in turn, to novel sequencing and bioinformatic technologies to analyze the whole biochemical information conveyed by a given environmental sample; for instance, microbial communities in water samples from an abandoned mining site were analyzed using this powerful approach, revealing several metal transporters, TFs, and enzymes associated with the HM stress response by endogenous HM-tolerant cyanobacteria [82]. In another example, a metagenomics analysis of the microbiota of a swine lagoon wastewater by high-throughput sequencing unfolded successful removal of nitrogen and phosphorous by *Chlorella* sp., as well as interesting pieces of information on the interaction of microbial communities within that ecosystem, which have led to improvements in the phycoremediation efficiency of microalgae [83]. Therefore, meta-omics analysis provides a snapshot of the response of a whole ecosystem to environmental changes and may ultimately provide a collection of target genes, useful for the design of novel microalgae strains with desired bioremediation capacities [84].

Despite the progress undergone by genetic tools to manipulate microalgae (see Table 1), a gap still exists between our understanding of the cellular mechanisms adopted by microalgae to survive under toxic concentrations of HMs, and application of the existing molecular tools to accordingly modify the cellular characteristics of given microalgal strains. In fact, detailed and comprehensive knowledge on growth, structure, metabolism, and functions in microalgae cells, upon exposure to metal stress, is a *sine qua non* to enable the effective engineering of those cells toward applicability in bioremediation. Furthermore, genome sequencing of newly discovered microalgal strains, as well as the establishment of strain-specific molecular tools, is crucial for effective genetic manipulation of such strains.

## 4. Genetic Engineering Targets to Improve Microalgal HM Bioremediation Capacity

### 4.1. Metal Transportation

As specialized gates in the cell membrane, metal transporters play an nuclear role in maintaining intracellular micronutrient homeostasis, by controlling the flux of metals in and out of the cell and organelles, while retaining the relative osmotic balance between the two membrane sides [85]. At least eleven unique gene families in the genome of microalgae have been characterized that encode metal transporters, and which exhibit a surprising similarity to those of yeasts, while a few transporter families are shared with plants, animals, or bacteria [24,44,86].

In *C. reinhardtii*, metal transporters have been classified into two main groups. Group A transporters, including natural resistance-associated macrophage proteins (NRAMP), zinc-regulated transporters (ZRT), iron-regulated transporters (IRT), Zrt-Irt-like proteins (ZIP), and Fe- (FTR) and Cu-transporter (CTR) families, ensure metal trafficking from the extracellular environment to the cytosol (as HM uptake), and from the cytosol into the vacuoles (as HM storage) [87]. Conversely, group B transporters, including members of the families of cation diffusion facilitators (CDF), P1B-type ATPases, FerroPortiN (FPN) and the Ca^2+^-sensitive cross-complementer1/Vacuolar iron transporter1 (Ccc1/VIT1), reduce cytosolic metal concentration via active efflux of metal ions and organometallic complexes into the extracellular surroundings, should metal concentrations exceed cellular requirements [12]. NRAMP transporters utilize the transmembrane proton gradient to mediate the transport of divalent cations to the cytoplasm [88]. Overexpression of NRAMP1 was reported in *Auxenochlorella protothecoides* under a high concentration of Cd in the medium [89]. Similarly, the upregulation of gene encoding NRAMP1, ZIP, and CTR transporters in *Dunaliella acidophila* was reported to improve Cd uptake [90]. DMT1, a divalent metal transporter from the NRAMP family, has been characterized as a key transporter of Mn, Fe, Cd, and Cu, across the cell membrane of *C. reinhardtii* and several other species [88,91]. The role of ZIP transporter genes in uptaking and sequestering Cd and Hg has also been demonstrated in *C. reinhardtii* [89,92]. Furthermore, genes that code for phosphate transporters (PTA) and aquaglycoporin (AQP) have been observed to increase As uptake in *Chlamydomonas eustigma* and *Microcystis aeruginosa* [93,94].

Despite their great potential, very few attempts have been made to improve HM phycoremediation through metal transporter engineering. Ibuot et al. [95] overexpressed CrMTP4, a metal-tolerant protein (MTP) from the Mn-CDF clade of cation diffusion facilitator family of metal transporters, in *C. reinhardtii*; marked increases in resistance to Cd toxicity, and in bioaccumulation efficiency due to increased transfer to and storage of Cd in acidic vacuoles were found. However, those authors emphasized that said single genetic modification failed to increase HM bioremediation capacity of the transformants over that of three wild-type, wastewater-adapted microalgae, viz. *Chlorella luteoviridis*, *Parachlorella hussii*, and *Parachlorella kessleri*. Further analysis revealed that the unique performance of the latter is due to higher antioxidant activities, including increased ascorbate peroxidase and carotenoid accumulation, as well as higher abundance and activity of other metal transporters [95,96]. Among five MTPs found in the genome of *C. reinhardtii*, MTP1 is believed to be localized in the vacuolar membranes—where it plays a crucial role in Zn homeostasis and Cd detoxification [87].

P1B-type ATPases—also known as heavy metal ATPases (HMAs)—are a class of metal transporters present across all taxa, including higher plants and macroalgae; HMAs play a critical role in metal trafficking across cell membranes [97,98]. Ibuot et al. [95] heterologously overexpressed AtHMA4—a plant Cd and Zn transporter from *Arabidopsis thalian*—in *C. reinhardtii*, and recorded increases in uptake and bioaccumulation of Cd and Zn by the transformed microalga. By overexpressing AtHMA4, either as a full-length protein or only as its C-terminal tail, they additionally showed that the reported increase was primarily the result of enhanced metal binding, rather than metal transport [99]. Similar results were attained following the heterologous expression of AtHMA4 in yeasts [100,101]. The cytosolic C-terminus of this protein contains a number of di-cysteine and histidine residues that mediate the high-affinity binding of Zn and Cd ions [102,103]. Ramírez-Rodríguez et al. overexpressed an arsenic hyperaccumulator, Acr3, which was localized in the vacuolar membrane; it acts as an efflux pump, and leads to a 1.5- to 3-fold increase in As removal capacity, as compared to wild type [104]. Furthermore, two highly conserved vacuolar proton pumps—vacuolar proton-ATPase (V-ATPase) and vacuolar proton pyrophosphatase (V-PPase)—generate the energy necessary to transport most solutes into the vacuoles [10]. Inhibition of V-ATPase activity by bafilomycin A1 (BFA1) in the diatom *Phaeodactylum tricornutum* caused upregulation of the genes involved in Ca signaling, sulfur metabolism, cell cycle, glycolysis, pentose phosphate pathway, porphyrin, chlorophyll metabolism, and lipid catabolism, as well as downregulation of the genes involved in ion transmembrane transport, ubiquitin-mediated proteolysis, SNARE interactions in vesicular transport, and fatty acid biosynthesis [105]. Moreover, expression of a universally expressible plasma membrane H^+^-ATPase (PMA) in *C. reinhardtii* led to a 3.2-fold increase in photoautotrophic production, under the high CO_2_ concentrations of (toxic) flue gas; this piece of evidence further highlighted the great potential of efflux pumps in microalgal bioengineering [106].

Given the requirement for such thiols and thiol-containing compounds as GSH and PCs, posed by cellular defense against HM toxicity, sulfur metabolism plays an imperative role in microalgal HM mitigation. The upregulation of biotin biosynthesis genes, which are involved in sulfur metabolism, and S-transporter genes was reported in *C. reinhardtii* when exposed to Hg^2+^ [92]. Genes involved in S-assimilation pathways, including those encoding methionine synthase (*mete*) and sulfite reductase (*sir1*), were also found to undergo upregulation in *C. reinhardtii* under HM stress. Additionally, overexpression of amino acid transporter genes in *C. reinhardtii* has been linked to an increase in Cd detoxification [107]. Other known HM transporters, well-characterized in plants but less so in microalgae, include multidrug resistance-associated proteins (MRP), ABC transporters of mitochondrion (ATM), pleiotropic drug resistance (PDR) transporters, yellow-stripe-like (YSL) transporters, and Ca^2+^ cation antiporters (CAX) [53].

Proteomic analysis of high HM tolerance and accumulation in *Euglena gracilis* unfolded a significant increase in expression of the major facilitator superfamily (MFS) transporters, cadmium/zinc-transporting ATPase, and HM transporting P1B-ATPase, as well as metal-binding, thiol-rich proteins, following HM exposure. A major MFS transporter involved in HM compartmentalization in cellular organelles experienced a 5.5-fold increase in expression level upon presence of HMs in the medium. Two P1B-ATPases, HMA2 and HMA3, known for their role in HM efflux, in and out of the cell and vacuoles, respectively, were also upregulated by ca. 3.6-fold, and further claimed to be key mediators of metal homeostasis in HM-exposed microalgae. In addition, a transmembrane TrkA transporter, involved in potassium transport and sodium/sulfate symport, showed a 6.5-fold increased expression once exposed to HM [108].

The feasibility of expressing bacterial metal transporters in plants has been widely demonstrated [109,110]; this approach also appears to be feasible in attempts to increase phycoremediation capacity in microalgae. Heterologous overexpression of metal ion/H^+^ antiporters CAX2 and CAX4, from *A. thaliana* in *Nicotiana tabacum,* increased uptake and sequestration of Cd, Zn, and Mn by 70–80% in transgenic plants, as compared to wild type [111,112]. Similar results were reported when a vacuolar ZAT Zn transporter from animal origin was overexpressed in *A. thaliana* [113]. Such ABC transporters as MRP2 were shown to be significantly upregulated; they increased HM uptake and sequestration in the vacuoles of *C. reinhardtii* and *D. acidophila* cells [92,114,115]. An ABC-type YCF1 transporter has been identified in yeasts and plants as a vector for transport of cytosolic GSH-complexed Cd into vacuoles [116]. Heterologous expression of the *YCF1* gene in *A. thaliana* yielded transgenic plants with increased Cd and Pb tolerance [117]. In another study, the human multidrug resistance-associated protein (hMRP1) gene—encoding an ABC-type multidrug resistance-associated transporter—was overexpressed in *N. tabacum*; the transformants exhibited higher tolerance against Cd when compared to wild type. Notably, hMRP1 is a well-known protein involved in the multidrug resistance of cancer cells, where it performs an efficient efflux of a wide range of cytotoxic compounds, including HMs [118]. Endogenous ABC transporters from the MRP subclass have also been characterized in microalgae as key mediators of metal homeostasis. Among seven MRPs identified in the genome of *C. reinhardtii*, four are glutathione S-conjugate pumps present in the vacuolar membrane, and able to transport metal-GSH complexes into the vacuoles [85].

ATM/HMT are half-size ATP-binding cassette transporters, and located either in the vacuolar membrane or the mitochondrial membrane of microalgae; among them, Cds1 is known to play an important role in Cd tolerance, by facilitating export of Cd from mitochondria [87]. HMT1 was characterized in *Saccharomyces pombe* to be a vacuolar transporter capable of internalizing HM-MT complexes in the yeast vacuole [119,120]. Furthermore, ATM/HMT2 and ATM/HMT3 are localized in the mitochondrial and vacuolar membranes, respectively, where they mediate sequestration of Cd-phytochelatin complexes [29].

Despite the major efforts presented above, many microalgal and plant HM transporters remain to be identified at molecular level, and characterized in terms of localization, transport features, and specificity. Studies in this regard are, thus, crucial for a deeper understanding of the pathways followed by HM trafficking and accumulation in microalgae [1].

### 4.2. Metal Chelation

Extensive evidence pertaining to microalgae under metal stress confirms the overexpression of a number of metal-binding organic molecules, as a key strategy elected thereby to reduce the toxic effects of HMs, and handle them in the form of a chelated complex, toward storage in the organelles or plasma membrane. By sharing their free electrons and binding in a chelation process, HMs have their extreme reactivity confined, so the associated cell oxidative stress is minimized [5].

MTs are a group of genetically-encoded (class I and II), or enzymatically-synthesized (class III), polypeptides, ubiquitously found in living organisms and playing an important role in metal homeostasis and trafficking [121,122]. They contain a few aromatic residues (<10%), but a high proportion (15–35%) of cysteine and, to a lesser extent, histidine residues; such structure accounts for the high metal-binding capacity of MTs [123,124]. The high variability observed in the amino acid sequence of MTs—even among closely related organisms—implies a previous active evolutionary change, responsive to environmental conditions and cellular signals [5].

Class I MTs consist of two smaller Cys-rich domains, and a large spacer region in between. Class II MTs are low-molecular-weight (6–7 kDa) proteins, possessing three Cys-rich domains, separated by 10–15 residues; they are located in the cytosol, and mainly involved in the control of intracellular concentrations of metals at regular levels. Class III MTs—also known as phytochelatins (PCs)—are enzymatically synthesized thiol-containing oligopeptides; they are typically composed of three amino acid residues, *viz.* γ-Glu, Cys, and Gly [125].

The biosynthesis pathway of PCs starts with the formation of γ-glutamylcysteine from cysteine and glutamic acid, catalyzed by glutamate–cysteine ligase (also referred to as glutamylcysteine synthetase). Glutamylcysteine is next ligated with glycine by GSH synthetase, thus, forming GSH, which acts as the main redox buffer in eukaryotic cells, meant to protect them against oxidative and metal stresses. While GSH is able to chelate metallic cations through the thiol group of the side chain of Cys, it can also attach to other γ-glutamylcysteine units aided by phytochelatin synthases to eventually form PCs [126,127]. Enhanced activity of the enzymes involved in phytochelatin biosynthesis, along with a marked increase in the supply of GSH and PCs upon exposure to HMs, have been well-documented for several microalgae species [128,129,130,131,132,133,134,135,136,137]. Two GSH biosynthesis enzymes, γ-glutamylcysteine synthetase (encoded by *gshI*) and glutathione synthetase (encoded by *gshII*) from *E. coli*, were overexpressed in *Brassica juncea*; this manipulation led to a 25% increase in Cd uptake in transgenic plants versus wild type [138]. Recently, overexpression of a synthetic gene (*gshA*) encoding for γ-glutamylcysteine synthetase was shown to significantly increase Cd tolerance of *C. reinhardtii* [138,139]. Furthermore, the *CrGNAT* gene, encoding an acetyltransferase involved in histone methylation and chromatin remodeling, was overexpressed in *C. reinhardtii*; under toxic concentrations of Cu, a marked increase in cell population, chlorophyll accumulation, and photosynthesis efficiency was observed compared to wild type, while *CrGNAT* knockdown lines with antisense exhibited sensitivity to Cu stress. Further analyses revealed that acetyltransferase may play a key role in inducing the accumulation of GSH, MTs, and PCs in microalgae [140]. In one study, phytochelatin synthetase from wheat was overexpressed in tobacco and the transgenic plants were able to produce 100-fold biomass in HM-contaminated soils, when compared to hyperaccumulator *Thlaspi caerulescens* [141]. Increased expression of phytochelatin synthase was found to improve cellular tolerance to heat, salt, carbofuran, and UV stresses [142]. Additionally, it was suggested that targeting phytochelatin synthase, to such a specific organelle as the chloroplast, induces sensitivity, and to the cytosol induces tolerance against As stress [143].

While the structure and function of plant MTs have been thoroughly investigated [47,144,145], similar reports on microalgal MTs—with the notable exception of ciliates [146,147]—are scarce [5,49,125,148]. Cai et al. [149] authored the first report on the heterologous expression of MTs in microalgae; overexpression of a chicken MT-II in cell wall-deficient mutants of *C. reinhardtii* increased tolerance to Cd, and enhanced sequestration thereof by about two-fold in the transgenic microalgae. In another study, a fusion protein composed of low CO_2_-induced plasma membrane protein and MT-II polymer was expressed in *Chlamydomonas* sp., which led to a five-fold increase in Cd uptake by transformants relative to wild type [24]. These authors also evaluated the metal recovery capacity of transgenic microalgae, retrieved from contaminated sediments, using in situ sonication, and found that it was twice that of its wild counterpart [150]. Proteomic analysis of the effect of exposure to HM upon *E. gracilis* unfolded several proteins possessing metabolic roles that contribute to the microalgal response to HM stress. These included glutathione synthetase (with 14-fold increase), γ-glutamylcysteine synthetase (2.5-fold increase), cysteine desulfurase (*ca.* 2-fold increase), glutathione transferase (2.6-fold increase), mitogen-activated protein kinases (4.5-fold increase), heat shock proteins and chaperones (up to 11-fold increase), sulfate transporter ThiS (2.8-fold increase), leucine-rich repeat extensin-like protein, involved in cell wall biosynthesis (15-fold increase), and various antioxidant enzymes.

Among 98 different types of metal-binding proteins with increased expression, when exposed to HM, MTs were surprisingly not detected in *E. gracilis* cells [108]. The heterologous expression of phytochelatin synthase (OAS-TL or PCS) from *A. thaliana* in *Mesorhizobium huakuii,* increased Cd accumulation in this transgenic bacteria by 25-fold [109]. In another study, the constitutive overexpression of cysteine synthase (encoded by Atcys-3A) in *A. thaliana,* increased cellular cysteine and GSH levels, and the transformants accumulated 72% more metal compared to wild type [151]. Phytochelatin synthase from *Spinacia oleracea* was likewise overexpressed in *N. tabacum*; the transgenic plants exhibited a significant improvement in both Cd and Ni tolerance and accumulated HMs 2.8-fold compared to wild type [152].

O-acetyl-l-serine is known to be a substrate for OAS-TL, and one of the precursors in GSH biosynthesis; it is produced by reaction between l-serine and acetyl-CoA, catalyzed by serine-O-acetyltransferase (SAT). Overexpression of mitochondrial SAT of *Thlaspi goesingense* induced GSH accumulation in leaves of *A. thaliana*, and also increased tolerance to several HMs [153]. Furthermore, adenosine phosphosulfate from *A. thaliana* was overexpressed in *B. juncea* seedlings; the corresponding transformants displayed a two-fold increase in GSH content, and a substantial increase in tolerance to, and accumulation of, several HMs [154].

MT genes have also been reported in *Synechococcus*, as well as in seven other blue-green microalga strains [30]. It has been suggested that oligopeptide chain length and cysteine residue distribution determine the capacity of MTs and their host cells for HM binding and remediation. Some microalgae strains, bearing higher HM tolerance, appear indeed to synthesize MTs with longer chain length and more frequent cysteine residues. This realization may be taken advantage of to engineer microalgae with improved HM removal capacity, via heterologous expression of enzymes for synthesis of long cysteine-rich MTs, from hyper-tolerant species, in some transgenic strains [155,156].

Orthophosphate polymers—also known as polyphosphates (polyP)—have been implicated with accumulation of HMs in both prokaryotic and eukaryotic organisms [157,158]. Biosynthesis of polyP in microalgae is regulated by the activity of exopolyphosphatase, or else through compartmentalization mechanisms, mainly with the contribution of acidocalcisome membrane transporters [159]. The functions performed by polyP in microalgae include cycling phosphorus in the ocean, acting as a phosphorus reservoir in the cell, and providing cellular defense against nutrient, osmotic, thermal, and HM stresses [159,160]. Consequently, polyP formation facilitates HM sequestration and storage and may regulate chelation and compartmentalization of such toxic ions [44,161]. PolyP synthesis in prokaryotes is catalyzed chiefly by (reversible) ATP-specific kinases, PPK1 and PPK2, using GTP as a substrate [162]. Overexpression of PPK1 in cyanobacterium *Synechococcus* doubled its polyP content [163]. Most genes and proteins associated with polyP biosynthesis in eukaryotes remain unknown, while overexpression of prokaryotic PPK1 was found toxic for yeast and plant cells [164,165]. Instead of PPK, the vacuolar transporter chaperone (VTC) complex appears to be responsible for polyP synthesis in *Chlamydomonas*, whereas exopolyphosphatases (PPX) are the key enzymes responsible for its degradation [166]. The important role played by polyP in microalgal HM sequestration justifies examining how genetic engineering will affect the (characterized) genes; identification of other genes and enzymes associated with polyP metabolism might allow effective manipulation of said key cellular component for bioremediation purposes. It must be noted, however, that changes in phosphorous level—as one of the most critical nutrients in microalga cultivation—may have unpredictable and disturbing effects upon P homeostasis in the cell.

Amino acids are known to function as major players in cellular defense against metal and oxidative stress. Under toxic levels of Cd and limitation of N, *C. vulgaris* increased its ability to accumulate ketogenic and glucogenic amino acids, as well as such metal-binding amino acids as proline, histidine, and glutamine [167]. Proline behaves as a signaling molecule; its metabolic roles include regulation of intracellular osmotic pressure, prevention of protein denaturation, maintenance of membrane integrity, stabilization of enzymes, and quenching of toxic ROS, under various biotic and abiotic stress conditions, in both plants and microalgae. Furthermore, Pro accumulated in the cytosol contributes to alleviate metal stress via chelation of HMs and regulation of water potential [168,169,170].

A mothbean pyrroline-5-carboxylate synthase (P5CS), and a fusion protein composed of chicken MT-II and a plasma membrane protein were separately overexpressed in *C. reinhardtii*. The MT-expressing microalgae showed significantly enhanced tolerance to toxic concentrations of Cd; and its Cd-binding capacity increased by 2- to 5-fold compared to wild type. Furthermore, the P5CS-expressing microalgae produced 80% more free Pro and showed a 4-fold increase in its Cd-binding capacity. Proline was accordingly claimed to contribute to HM tolerance by enhancing GSH and PC biosynthesis, as well as reducing free radical damage, via physical quenching of oxygen singlets and chemical reaction with hydroxyl radicals [171].

On the other hand, *N. tabacum* transformants, expressing a fragment of proline dehydrogenase gene (in antisense orientation) from *Arabidopsis,* exhibited higher Pro content, and elevated osmotic pressure and salinity resistance, besides higher tolerance to Pb, Ni, and Cd [172]; similar results have been reported for other amino acids. Overexpression of the *HISN3* gene—encoding phosphoribosylformimino-5-aminoimidazole carboxamide ribonucleotide isomerase—induced a moderate increase in His accumulation, and significantly enhanced Ni tolerance in transgenic *C. reinhardtii* compared to wild type [173]. Similarly, *C. reinhardtii* cells were transformed with the *HAL2* gene, which regulates synthesis of Cys, leading to a five-fold increase in metal binding capacity of the transgenic microalgae [24]. Another amino acid osmolyte, glycine-betaine, was also shown to be overproduced by upregulation of serine decarboxylase (*SDC1*) in *C. reinhardtii* cells, under Cd stress [107].

As previously mentioned, such organic acids as oxalic, citric, tartaric, malonic, and malic can also chelate HMs; this complexation tends to occur in vacuoles, where acidic pH favors cleavage of HM-MT and formation of HM-organic acid complexes [10,174,175]. Aside from the intracellular presence of metal-binding organic compounds, evidence shows that these molecules may also be released by microalgae into their extracellular environment in response to stress [22,26,176,177]. Therefore, biotechnological approaches appear feasible to increase microalgal accumulation or release of such organic metabolites, with the goal of improving HM phycoremediation thereby.

### 4.3. Metal Biotransformation

Xenobiotic or endobiotic chemicals in a cell are metabolized to fewer toxic products by resorting to biotransformation. The detoxification pathways of HMs in microalgae consist of several enzymatic reactions, developed ab initio by the cell to reduce their toxic nature, by confining the metal ions to organic structures, where their oxidative potential is severely constrained [35].

In *C. vulgaris*, a chromate reductase (ChrR) has been characterized that converts Cr(VI) to Cr(III), via an enzymatic reaction, involving oxidation of GSH, which confers a high tolerance against Cr toxicity [178,179]. Two bacterial genes, *gsh1* and *arsC* (arsenate reductase) were overexpressed in *A. thaliana*, thus, resulting in transgenes bearing significantly higher tolerance to As(V) than wild type [180]. Moreover, an arsenate reductase (CrACR2s), found in *C. reinhardtii,* was shown to reduce arsenate to (less toxic) arsenite, with the extra electrons transferred to glutaredoxin [181]. In several microalgal strains, arsenic is biotransformed through various mechanisms—the most common being reduction of As(V) to As(III)—complemented by methylation of As(III) to monomethylarsonate (MMA), brought about by oxidase and S-adenosylmethionine (SAM), followed by conversion of MMA(V) to dimethylarsinate (DMA(V)), which is further reduced to DMA(III). Finally, DMA(III) is converted to a range of organoarsenicals, e.g., arsenolipids, arsenosugars, arsenobetaine, and arsenoribosides [182,183,184,185,186]. By the same token, the expression of mercuric reductase permits biotransformation of Hg^2+^ to elemental Hg and metacinnabar (β-HgS), in strains of microalgae *Selenastrum minutum*, *Chlorella fusca*, and *Galdiera sulphuraria* [187].

In attempts to improve the ability of *Chlorella* spp. DT to detoxify mercury, a bacterial mercuric reductase (*merA*) gene from *Bacillus megaterium* was overexpressed in this microalga; the resulting transformants exhibited a two-fold increase in Hg^2+^ bioremoval capacity compared to wild type [188]. The applicability of this method had been previously demonstrated in various plants, when enhancing their tolerance against mercury. Concurrent expression of both *merA* and *merB* genes, codon optimization, and targeting of MerB (organomercurial lyase) to the endoplasmic reticulum (ER), also proved effective to increase Hg^2+^ and R-Hg^+^ biotransformation in transgenic plants, by up to 10-fold [189,190,191,192,193,194,195,196,197,198]. Additionally, integration of the aforementioned two genes in the chloroplast genome of tobacco significantly expanded tolerance to phenylmercuric acetate and extent of mercury bioaccumulation in the transgenic plants [199,200]. Organomercurial lyase mediates the protonolysis of organic mercury to Hg^2+^, while mercuric reductase reduces Hg^2+^ to Hg^0^; hence, overexpression of cytosolic MerA and ER-located MerB, using (microalgae-specific) codon optimized genes might prove an effective approach to improve the HM-biotransformation capacity of microalgae [201]. Remember that the Mer operon also possesses the coding genes for three inner membrane transporters (MerC, MerT, and MerF), involved in the transport of mercury into the cytosol; periplasmic MerP is responsible for funneling metal ions (preferably ionic mercury) to those inner membrane metal transporters [202]. Therefore, expression of MerP, or a fusion of MerC/T/F to a metal chelator (as membrane-bound Hg^2+^ trap) will putatively improve the ability of the microalgae to bioremove mercury [201]. Given the environmental concerns over excessive release of Hg^0^, as volatile metabolites, into the atmosphere by such engineered species, it might be safer to sequester ionic mercury inside the cell via binding to a chelating molecule.

Another interesting strategy for HM bioremediation would be targeting the aforementioned mercury translocators to the chloroplast inner envelope membrane, allowing accumulation of HM in the chloroplast; however, toxic effects of such modification upon the photosynthetic apparatus and other vital structures of the chloroplast are to be evaluated in advance [203].

Misincorporation of selenocysteine (SeCys) and selenomethionine (SeMet) into proteins accounts for a biologically unfavorable effect of Se when present at toxic levels. Conversion of said compounds to non-protein amino acid methylselenocysteine (MetSeCys) by selenocysteine methyltransferase (SMT) has been described in Se-hyperaccumulating plants [204]. SMT was overexpressed in *A. thaliana* and *B. juncea*, and the transformants showed substantially increased levels of Se tolerance compared to wild type [205,206]; a similar strategy may potentially be employed to improve Se phycovolatilization in microalgae.

Cytochromes-P450 (CYPs) are as well recognized for their role in the enzymatic biotransformation of toxic molecules. A CYP-like protein from rice was heterologously expressed in *A. thaliana* and the transgenic plants exhibited significant tolerance against abiotic stresses, including toxic levels of HMs. This underlying gene was reported to help plant cells fight environmental stress by modulating auxin metabolism, defense mechanisms, hypocotyls growth, stomatal movement, cell elongation, cytokinesis, apoptosis, and light response. The improved tolerance and bioaccumulation of As and Cr were more specifically attributed to phosphate transporters and/or ABCC transporters, following qRT-PCR analysis of differential gene expression patterns, between transgenic and wild-type lines [207]. Other HM volatilization mechanisms described in microalgae include a photoreduction pathway, found in *C. vulgaris,* for the biotransformation of Cr [208], intracellular and extracellular biosyntheses of metal nanoparticles, and reductive interactions with functional groups of biomolecules in- and outside the cell [40,209,210,211]. Despite the improved efficiency and wide range of applications that HM-volatizing genes and proteins might offer to the phycoremediation process, the underlying pathways for biotransformation of HMs in microalgae (and plants) remain mostly unknown and, thus, still require extensive in-depth research.

### 4.4. Oxidative Stress Response Regulation

HMs induce the generation of superoxide radical (O^2−^), hydrogen peroxide (H_2_O_2_), hydroxyl radical (HO^−^), and singlet oxygen (^1^O_2_)—collectively known as ROS [96]. These free radicals interact with biologically active compounds, and eventually damage molecular and cellular structures, including transporters, enzymes, structural proteins, and membrane lipids.

The physiological, biochemical, and gene expression characteristics of *C. reinhardtii* were evaluated under toxic concentrations of Cu; inhibition of cell growth and photosynthesis, variation of total chlorophyll content, and marked increase in lipid peroxidation were accordingly observed [212]. Similarly, transcriptional analysis of *D. salina* and *C. reinhardtii,* exposed to Cd and Pb, respectively, revealed that the encoding genes of several antioxidant enzymes were upregulated in microalga cells subjected to HM stress [114,213]. Along with upregulation of antioxidant enzymes, the overexpression of thioredoxin (Trx), heat shock proteins (HSPs), and carotenoids was documented in *A. protothecoides*, *C. vulgaris*, and *C. reinhardtii* cells, in response to toxic levels of HMs [54,80,107]. A similar analysis on *Amphora coffeaeformis*, *Navicula salinicola*, and *D. salina* under HM stress unfolded a marked increase in antioxidant defense-related genes, a response shared by all three species of microalgae [214].

Further to metal chelation and phycovolatization, microalgae have developed a number of strategies to reduce the oxidative stress imposed by HMs. Well-known enzymatic antioxidants, e.g., superoxide dismutase (SOD), peroxidase (POD), catalase (CAT), guaiacol peroxidase (GPX), and glutathione-S-transferase (GST), actively convert superoxide radicals to hydrogen peroxide, and subsequently to water and oxygen, while such non-enzymatic antioxidants as Pro, ascorbic acid, and GSH may directly quench ROS via complexation [1,50,96]. Gene expression analysis of *C. reinhardtii*, under toxic concentrations of HMs, indicated that glutathione peroxidase plays a crucial role in oxidative defense, for protecting the thylakoid membranes from oxidative stress. This cytosolic enzyme, which catalyzes formation of a thiol bond between two GSH molecules and reduces peroxide radicals to their corresponding alcohols and oxygen, emerges as an interesting candidate for strain improvement purposes [215].

As part of an indirect mechanism, heat shock proteins (HSPs) act as molecular chaperones to protect and repair proteins under HM stress [216,217,218,219]. HSPs are highly conserved among microalgae and other organisms, and involved in transport, folding, unfolding, assembly, and disassembly of proteins, as well as degradation of misfolded or aggregated proteins. The role of HSPs, including HSP20, HSP70, and HSP100, in mitigating protein denaturation was illustrated in green microalga *Tetraselmis suecica*, following HM stress [220]. Therefore, improvement of the reducing potential, and consequently phycoremediation capacity, appears possible by genetically inducing overproduction of enzymatic and non-enzymatic antioxidants in the microalga cells.

### 4.5. Metal Stress Response Regulation

Cellular response to HM stress, and consequent detoxification mechanisms, are mainly regulated by key components in the metal regulatory network held by microalgae. Several regulatory molecules contribute in the control of the HM-detoxifying factors, via genome-wide changes in gene expression; this leads the cell, in turn, toward a particular biochemical state that minimizes the adverse effects of HMs. The role of TFs, phytohormones, and miRNAs will be discussed below, for being the master regulators of metal stress response in microalgae.

TFs are DNA-binding proteins, which interact with enhancer or promoter sequences of a cluster of genes, to regulate their transcript levels in the cell [221]. Metal response element (MRE)-binding transcription factor-1 (MTF-1) is the main metal-sensing TF found in eukaryotes. Zn binding to its zinc finger domain reversibly and directly activates the DNA-binding activity of MTF-1. The activated MTF-1 is then transported to the nucleus, and assists histone acetyltransferase p300 in binding specific promoters, so as to induce or repress transcription [222]. Aside from high intracellular concentrations of Zn, MTF-1 can be indirectly activated by Cd or Cu, as a result of the oxidative stress triggered by HMs. The genes upregulated by MTF-1, including *Znt1* and *Znt2* (zinc efflux transporters) [223], *Zip10* (zinc influx transporter), *Gclc* (glutamate-cysteine ligase catalytic subunit), *Ndrg1* (N-myc downstream regulated 1), *Sepw1* (GSH-binding selenoprotein), *TXNRD2* (thioredoxin reductase 2), *FPN1* (FerroPortiN 1), and *Csrp1* (cysteine- and glycine-rich protein 1), have all been reported to contain multiple copies of MRE motif 5′-TGCRCNC-3′ in their UTR; hence, the latter seems to be an MTF-1-binding *cis*-regulatory element [224]. Furthermore, it was demonstrated that MTF-1-dependent activation of MT gene promoters requires the presence of zinc-saturated MTs in a cell-free transcription system, whereas thionein (the metal-free form of MT) inhibits activation of MTF-1 [225].

In line with previous studies on metal stress-specific TFs in plants and microalgae, it appears that some of these regulatory proteins, including C2H2, AP2, MYB, bHLH, and YABBY, are part of the mechanism of tolerance to HM stress; this occurs due to enhancement in activity of enzymatic antioxidants, increase in production of malondialdehyde (MDA), and sequestration of HMs. On the other hand, bZIP, SBP, and HB TFs have been found to play regulatory roles in the uptake and accumulation of HMs [226]. Transgenic tobacco and petunia plants, expressing RsMYB1 TF, which controls regulation of anthocyanin, were found as more stress-tolerant than the wild type under toxic levels of Cd. Gene expression analysis revealed that RsMYB1 overexpression led to increased expression of genes associated with metal detoxification (GST and phytochelatin synthase) and antioxidant activity (SOD, CAT and POX), thus, contributing to significantly boost cellular defense in plants against abiotic stress [227].

WRKY13 was claimed as another metal stress-related TF in *A. thaliana*, where it activates transcription of PDR8, an ABC transporter involved in Cd extrusion. Overexpression of WRKY13 led to a decrease in Cd accumulation and enhancement in Cd tolerance of transgenic plants, whereas WRKY13 loss-of-function mutants exhibited increased accumulation of Cd and sensitivity thereto [228]. Hence, such putative TFs as WRKY13, or such transporters as PDR8, involved in the regulation of HM extrusion, could allow for the design of transgenic microalga cells with lower metal extrusion ability and enhanced HM accumulation. Note, however, that the cellular stress conferred by the resulting hyperaccumulation of HMs must be balanced, in parallel with overproduction of components of metal detoxification mechanisms (e.g., metal chelators and vacuolar metal transporters) in transgenic microalgae.

Recently, a copper response regulator (CRR1) was claimed to be a TF that controls expression of over 60 genes in *Chlamydomonas* spp., including those associated with accumulation of plastocyanin and cytochrome c6, as well as Cu homeostasis. CRR1 is activated in response to Cu deficiency and, thus, induces the expression of several metal transporters and redox enzymes, which may increase Cu uptake, while protecting microalgae against subsequent stress [229]. SbMYB15 has also been reported as a MYB TF, with a potential role toward HM tolerance. While the transcript level of *SbMYB15* increased by over 5-fold in *Salicornia brachiata* when in the presence of Cd and Ni, the constitutive overexpression of this TF increased growth and chlorophyll content in transgenic tobacco under toxic levels of HMs. The SbMYB15-overexpressing plants also exhibited low uptake of heavy metal ions and increased antioxidative activity compared to wild type [230].

Zhang et al. [231] reported that the basic region/Leu zipper TF, abscisic acid-insensitive5 (ABI5) is involved in ABA-repressed Cd accumulation in *A. thaliana*. Through physical interaction, ABI5 activates (the previously uncharacterized) MYB49 TF, which, in turn, upregulates bHLH38 and bHLH101 TFs; this leads to activation of iron-regulated transporter1 (IRT1) and two heavy metal-associated isoprenylated proteins, thus, increasing Cd uptake and accumulation. It was also reported that overexpression of MYB49 TF enhanced Cd tolerance in plant cells, while its disruption reduced the Cd bioremediation capacity of mutant cells. In a microarray analysis of 85 bHLH coding genes in *Cicer arietinum*, subjected to Cd and Cr stress, Yadav et al. [232] reported 10 hub genes from the bHLH family of TFs able to play potentially significant roles in regulating HM stress response. Heterologous overexpression of the OBP3-responsive gene *GmORG3*—a bHLH TF (ORG)—increased Cd tolerance and stabilized Fe homeostasis in transgenic soybean and tobacco, by specifically reducing phytotoxic effects induced by Cd stress and Fe deficiency [233].

In another study, the main downstream responses of *C. reinhardtii* to Pb toxicity were analyzed, and 20 putative TF genes associated to Pb tolerance, including C2H2 (C2H2-type zinc finger TF), AP2 (activator protein 2), MYB (myeloblastosis), bHLH (basic-helix-loop-helix), bZIP (basic region leucine zipper), SBP (SQUAMOSA promoter binding proteins), YABBY, GATA, and HB (homeobox), were comprehensively characterized. Differential expression of 67 genes, putatively related to hormones, unfolded the important role of hormone signaling upon the regulation of microalgal response to HM stress, while the overproduction of chelators and transporters was among the most pronounced metabolic changes, following metal toxicity [114].

Phytohormones are signaling molecules, bearing a wide array of cellular functions in higher plants and microalgae, and aimed at retaining growth plasticity during development. The role of phytohormones in harmonizing the cellular response to HM toxicity (and other abiotic and biotic stresses) has been well-documented [56,234,235,236]. Cytokinins (CKs), gibberellic acid (GA), auxins, abscisic acid (ABA), brassinosteroids (BRs), jasmonic acid (JA), ethylene (ET), and salicylic acid (SA) are the main classes of phytohormones. Although their exact mechanisms of action are mostly unknown, they have been claimed to prevent degradation of photosynthetic pigments, monosaccharides, and proteins and activate antioxidant defense responses required to sustain the growth of microalgae under stress conditions [56,234,237].

Interestingly, the exogenous application of phytohormones has proven effective to improve HM tolerance in microalgae. For instance, CK treatment was reported to improve survival rate of *C. vulgaris* and *A. obliquus* cells against Cd and Pb toxicity, by stimulating the activity of enzymatic antioxidants, as well as GSH, ascorbate, and PC biosynthesis [235,238]. Stimulating microalgae with exogenous GA and auxin was shown to mitigate HM stress by regulating photosynthesis, besides fatty acid and antioxidant metabolism [56,237,239]. Similarly, ABA and Br alleviated Pb, Cd, and Cu stress in *A. obliquus* and *C. vulgaris* cells, via induction of PC biosynthesis [238,240,241]. Furthermore, ET and SA effectively scavenged and detoxified ROS in *C. vulgaris* and *Haematococcus pluvialis* under HM stress, by inducing Pro/astaxanthin and SOD/CAT encoding genes [242,243,244]. When exogenously applied, auxin, CK, GA, and polyamine-spermidine (Spd) enhanced HM tolerance in *C. vulgaris* subjected to HM stress, by inhibiting heavy metal biosorption, restoring microalgal growth and primary metabolite level, and inducing the accumulation of antioxidant enzymes, ascorbate, and GSH. However, microalgae treated with JA underwent enhanced HM toxicity, increased metal biosorption and ROS generation, and exhibited a marked decrease in cell number, chlorophylls, carotenoids, monosaccharides, soluble proteins, ascorbate, antioxidant activity, and GSH content [237].

In view of the above facts, attempts to improve the bioremediation capacity of microalgae should rationally resort to optimization of their phytohormone profiles via genetic engineering approaches. For example, overexpression of a CK biosynthetic gene (IPT) in tobacco increased the transcript level of an MT-like gene [245]. On the other hand, mutations in *ipt1*, *ipt3*, *ipt5*, and *ipt7* genes, associated with CK biosynthesis in *A. thaliana,* led to enhanced Se tolerance, via a significant increase in the activities of CAT, ascorbate peroxidase, and glutathione peroxidase [246]. Similarly, a CK degradation enzyme, CKX1, was overexpressed to generate CK-deficient *Arabidopsis* and tobacco plants, and the resulting mutants exhibited higher accumulation of thiol compounds, thus, leading to improved tolerance against As stress [247]. Aside from photorespiration in *C. reinhardtii* [248], carbon metabolism in *C. vulgaris* [249], and photosynthesis in *Gracilaria caudata* [250], CKs were shown to regulate the cell oxidoreduction state [237].

GAs seem to be involved in HM stress tolerance in microalgae, via their effect upon photosynthesis pathways and ROS networks. GA-treated *C. vulgaris* showed higher HM biosorption capacity, along with increased fatty acid and lipid accumulation, which further boosted tolerance of said microalga to HM [251,252,253]. Auxin has been reported as mainly involved in inducing enzymatic and non-enzymatic ROS detoxification systems, when subjected to HM stress [56]. ABA mitigates the toxic effects of HMs, and functions as a central cross-talking agent among other phytohormones. While Pb exposure increased the intracellular level of ABA by 111% in *A. obliquus*, exogenous application of other phytohormones (i.e., CKs, auxins, and BR) improved HM tolerance, but decreased endogenous ABA level [56]. In *Scenedesmus quadricauda*, ABA improved cell growth by 2.1-fold, and induced the accumulation of saturated fatty acids by 12% under nitrogen starvation [254]. Similar results were reported for *Chlorella saccharophila*, *E. gracilis*, and *C. saccharophila*, following ABA treatment under HM stress [255,256].

BRs have been shown to increase growth rate, as well as contents of metabolites (viz. proteins, chlorophylls, and monosaccharides) and antioxidants in microalgae, thus, improving HM tolerance [257]. Furthermore, the role played by ET, JA, and SA upon HM tolerance in microalgae apparently includes upregulation of antioxidant enzymes, astaxanthin, and Pro, so as to minimize the oxidative stress caused by HMs [56]. Based on these data, phytohormones are claimed to be among the main regulators of HM tolerance in microalgae and, accordingly, consubstantiate an interesting target for phycoremediation-related strain improvement trials. With few exceptions, the coding genes for phytohormone biosynthesis remain essentially uncharacterized in microalgae [258]; hence, further omics analyses are warranted to characterize algal genes associated with phytohormone biosynthesis, and map endogenous hormone signaling networks in microalgae.

Several decades of extensive research on molecular configuration of biological systems has indicated that a large network of microRNAs (miRNAs) regulate the expression pattern of genes in the cell, in parallel to TFs and hormones. These small, noncoding RNAs form specific secondary structures that enable them to bind target mRNAs and this may lead to cleavage or repression of the translation of said mRNAs [259]. As in many other cellular processes, miRNAs play a key role in controlling HM stress response, mostly by regulating expression of the corresponding TFs [260]. Nonetheless, miRNAs involved in metal uptake and transport, sulfate allocation and assimilation, protein folding and assembly, metal chelation, antioxidant system, phytohormone signaling, growth/reproduction regulation, and miRNA biogenesis and action themselves, are also of importance for affecting HM stress response in microalgae [261,262,263].

Recent studies corroborate the interplay of hormones and miRNAs upon regulation of HM stress [264]. In plants, several stress-responsive miRNAs (e.g., miR395 and miR398) were found to be upregulated under toxic levels of Cd and Zn [265,266]; furthermore, miR397 and miR408 were characterized as extracellular metal chelators [267,268]. It was also demonstrated that several miRNAs exhibit time-dependent change in their expression pattern, suitable to regulate cellular response against HM stress [52,264]. Recently, transcriptomic analysis of *H. pluvialis,* under both excessive light and sodium acetate, revealed involvement of 434 miRNAs in the adaptive response of microalgae to abiotic stress [263]. Crucial regulatory roles of miR398, miR319, miR390, miR393, and miR171 have been pinpointed in the regulation of metal stress response in plants. Under high Cu levels, miR398 regulates induction of *CSD1* and *CSD2* mRNAs, which encode copper–zinc superoxide dismutase as vital scavenger of superoxide radicals [269]. Moreover, miR395, miR397, miR408, and miR857 were reported to control transcript abundance of laccase and PC genes under Cu stress, while miR390, miR319, miR528, and miR393 have been implicated in the auxin regulatory network [270,271,272,273]. After 24 h of treatment with Cd, microarray analysis of rice seedlings revealed that 19 miRNAs were differentially regulated, among which only miR528 (involved in miRNA biogenesis) was upregulated [274]. Transgenic *Brassica napus,* overexpressing miR395, showed a lower degree of Cd-induced oxidative stress upon Cd exposure, while chlorophyll, GSH, and non-protein thiol contents, as well as accumulated biomass and sulfur were higher in transformants compared to wild type [275]. In addition, miR808, miR396, miR390, miR319, miR160, and miR159 have been claimed to be involved in plant cell response against Al stress [276,277]. Given their relatively recent discovery, a huge research gap exists regarding discovery of metal-regulated miRNAs; hence, our understanding of the interplay between components of gene expression regulatory networks upon HM stress in microalgae remains quite limited.

### 4.6. Cell-Surface Bioengineering

As mentioned previously, adsorption of metal ions by the cell wall and other extracellular components in microalgae plays an important role in lowering the toxic effects of HMs. The microalgal cell wall alone is estimated to have a metal binding capacity of ca. 0.10 g_metal_/g_CDW_ [12,278]. This figure has been confirmed when cell wall-deficient mutants of *C. reinhardtii* were compared to wild type cells, in terms of Cd tolerance; survival rate of wall-less mutants was indeed 25–35% lower [149].

It was recently suggested that the metallosorption properties of the cell surface can be effectively modified to improve HM phycoremediation capacity and specificity of microalgae, in what has been termed cell surface engineering, or cell surface display [1,279]. This is possible by expressing metal-binding proteins (e.g., MTs and PCs) fused with an anchoring motif on the cell surface [280,281]. He et al. [150] expressed a membrane-anchored MT polymer in *C. reinhardtii,* using said approach, and reported a marked increase in Hg removal capacity of the transgenic microalga versus its wild type.

Given their relatively easy manipulation and rapid growth, yeasts and bacteria offer a rich source of experimental data on cell-surface engineering applications, which may then be used as a road map to conduct similar experiments in microalgae. Kuroda et al. [282] expressed a fusion protein of a His hexapeptide with the C-terminus of the sexual adhesion glycoprotein α-agglutinin (AGα1Cp), and an anchor attachment signal sequence on the cell surface of *S. cerevisiae*. Such surface-engineered yeast adsorbed 3–8-fold Cu^2+^ ions and was more resistant to copper than its parent strain. Moreover, about half of the adsorbed Cu was easily recovered upon EDTA treatment, without disintegrating the cells. They further engineered the hexa-His-displaying yeast cells, so as to self-aggregate in response to binding and accumulation of Cu^2+^, by transforming them with GTS1, a putative zinc-finger TF, able to induce cell-aggregation, under control of the copper ion-inducible *CUP1* promoter from a yeast MT gene [283]. In another study [284], those authors compared the Cd-chelating ability that MT and hexa-His displayed on the surface of the yeast cell, and concluded that the former is more effective for adsorption of Cd^2+^, while fusion of both further increased Cd adsorption and recovery. The same research team still examined the potential of expressing tandem repeats of yeast MT on the cell surface; adsorption and recovery of Cd on the cell surface, and survival rate under Cd stress, were increasingly enhanced by increasing the number of MT tandem repeats [285].

A 5-fold increase in the Pb^2+^ biosorption capacity of *S. cerevisiae* was observed upon anchoring short metal-binding NP peptides (harboring the CXXEE metal fixation motif of bacterial Pb^2+^-transporting P1-type ATPases) to AGα1Cp on the yeast cell wall [286]. Moreover, a surface exposed MerR (a metalloregulatory protein bearing high affinity and selectivity toward Hg) enhanced the Hg^2+^ adsorption capacity of *E. coli* by 6-fold versus wild type [287]. The Hg removal capacity of transgenic *C. reinhardtii* was also expanded by expressing a membrane-anchored MT polymer [150]. Similarly, recombinant *E. coli* overexpressing MT fused to the outer membrane domain of a maltose transporter (LamB), and exhibited a 15–20-fold increase in Cd binding capacity compared to wild type [288]. Furthermore, overexpression of a fusion protein, composed of glutathione S-transferase and MT, increased Ni^2+^ accumulation in a transgenic bacterium by 3-fold [289].

Cell surface engineering technology has also been suggested for the recovery of precious metals from wastewater, for bearing lower costs, and improved selectivity for the target metal compared to conventional methods [290]. For instance, a mutant protein of *E. coli* Ni^2+^-dependent transcriptional repressor (NikRm) was shown to selectively bind uranyl ions (UO_2_^2+^) and, thus, significantly increase the recovery of uranium from aqueous solutions when on the cell surface of *S. cerevisiae* [291]. In addition, metal-responsive TFs, able to bind and dissociate metal ions, can be repurposed as metal-binding proteins on the cell surface. Based on this concept, molybdenum was successfully recovered from an aqueous solution to a 50% yield, by locating the C-terminal domain of *E. coli* molybdate-binding TF (ModE) on *S. cerevisiae* via the α-agglutinin-based display system [292]. Moreover, a single amino acid mutation significantly and selectively increased the binding of ModE to tungstate, and engineered yeasts displaying this mutant ModE exhibited preferential uptake of tungstate ions [293]. Given the wide range of ligands susceptible of display on the cell surface—including metal-binding moieties for bioremediation of HMs—cell surface engineering will likely play an important role as a biotechnological tool in the near future [294].

## 5. Discussion

Heavy metals pose a serious danger to ecosystems and, ultimately, to human health; however, the physicochemical methods currently in use for their removal from aquatic environments suffer from a number of shortcomings. Biological methods appear quite promising and microalgae, in particular, offer unique features, so that they may be considered as the next generation of biosorbents for the treatment of HM-contaminated wastewaters. These microorganisms use sunlight for energy and atmospheric carbon dioxide as a carbon source, besides a few minerals present in most wastewaters, and have proven useful in producing such valuable chemicals as biodiesel, biomethane, bioethanol, biochar, and antioxidants. Furthermore—unlike conventional methods of HM remediation—microalgae do not generate toxic sludge, are easy to culture and maintain, exhibit good binding affinities, and have the potential to significantly reduce processing costs. At the same time, microalgae reduce the organic and inorganic loads of wastewater, sequester carbon from CO_2_ (thus, reducing greenhouse effects), and release oxygen from water.

Despite the above advantages, microalga-mediated HM remediation is yet to be feasible at a large scale. One of the main obstacles is the low concentration attainable of microalgae, which, by itself, reduces process productivity. Hence, the first challenge is to develop microalgae strains bearing higher resistance against biotic and abiotic stresses, and concomitantly increased biomass yield in wastewater; several genetic engineering approaches have been discussed in this regard, in terms of success, or potential to improve HM phycoremediation capacity and specificity.

Despite the current gap in availability of genome sequences and/or editing tools for most microalgae species found so far, huge opportunities are anticipated from existing knowledge, pertaining to genetic and metabolic engineering of such model microalgae as *C. reinhardtii*, *C. vulgaris*, *D. salina*, *N. oceanica*, or *P. tricornutum*. Previous experience indicates that single gene mutations will not necessarily succeed in effectively improving HM bioremediation features. Therefore, a holistic approach appears necessary when designing genetically engineered microalgae for better performance.

Toward this goal, hundreds of previous studies, pertaining to characterization of metal transporters, metal chelators, metal volatilization enzymes, oxidative and metal stress response regulators, and cell surface engineering were hereby reviewed, in a comparative and critical fashion, with the ultimate goal of finding appropriate target genes, see Table 2. In parallel, computational and mathematical modeling approaches (e.g., FBA) might help, in order to avoid putative incompatibilities between multiple genetic modifications, consequently leading to microbial cell factories with optimized metabolic efficiency.

Based on the literature data, overexpression of the group A family of transporters (i.e., NRAMP, ZRT, IRT, ZIP, FTR, and CTR) may boost metal uptake by microalgae, and storage thereof in vacuoles. Although enrichment of the cell in MTs, PCs, Pro, GSH, and antioxidants by overexpressing their biosynthesis genes appears a logical step toward improved HM phycoremediation, such a minor change may not suffice to ensure the achievement of a robust microalgal strain. However, overproduction of specific antioxidants may offer the dual advantage of enhancing HM tolerance and reducing microalgal production costs, through a biorefinery scheme, entailing separation and commercialization of the antioxidant itself. It should be emphasized that overproduction of MT biosynthesis enzymes may prove ineffective if the intracellular GSH pool is exhausted, as a consequence of said overproduction. Expression of TFs, phytohormones, and miRNAs as master regulators of cellular response to metal toxicity, and subsequent oxidative stress, should instead have a deeper and broader impact upon a sound biophysicochemical state of the cell—able to let it thrive under such harsh environmental conditions. Expression of these transcriptional and translational regulators may result in whole-cell adjustment of its redox state, by affecting a number of metabolic pathways, including intracellular pools of antioxidants and metal-binding polypeptides.

Given the eventual limit for the number of chelated metals that can be stored within cytosol and membrane-bound organelles, transgenic microalgae with enhanced metal uptake and chelation abilities must also be armed with the enzymes needed to transform the stored metals into benign organometallic compounds. While a single enzyme capable of transforming all HMs has not yet been identified, overexpression of an enzyme for each metal will overcomplicate the genetic engineering process and may pose an excessive metabolic burden on the cell. Therefore, when designing tailor-made strains of microalgae for municipal wastewater treatment applications—where they will face a mixture of HMs at various concentrations—volatilization enzymes may be deemed redundant or inadequate. Conversely, such engineered microalga strains could be effectively used for bioremediation of industrial effluents containing high concentrations of specific HMs, as well as the deliberate recovery of precious metals, including seabed mining.

The cell surface remains in direct contact with the outer environment and acts as the first line of cellular defense against HM-mediated stress. Cell surface engineering will allow microalgae to adsorb and, thus, detoxify, higher amounts of HMs in a metabolism-independent manner; this simple step is expected to play an important role in the future of genetically enhanced phycoremediation. Overexpression of metal-binding proteins on the cell surface allows easy recovery of adsorbed species, increases cell flocculation ability, and greatly reduces the contact time required for effective phycoremediation of HMs. This is prone to lower the cost/energy associated with the harvesting of microalgal biomass—still one of the main challenges of wastewater phycoremediation—and would greatly improve process productivity. Selective removal of a particular metal is also possible by expressing the corresponding ligand on the microalgal surface. Furthermore, displaying tandem repeats of MTs anchored to cell wall- or membrane-bound proteins may appear as a possible approach to boost the HM adsorption capacity of microalgae.

Despite the above technical difficulties, environmental concerns over the release of engineered strains, and associated stringent regulations for their use, represent probably the major obstacle to develop genetically improved microalgae for bioremediation. A refreshed look at recent advancements of molecular tools, in their ability to minimize the probability of lateral gene transfer and at processing measures, to prevent release and/or survival of transgenic microalgae in the wild, seem necessary to deploy the full potential of such beneficial technology, as long as this is complemented by having regulatory guidelines and public feelings reshaped toward the inevitable use of transgenic microalgae. The use of dead biomass, or immobilization of live microalgae, in/on biopolymers, constitute possible solutions to address these issues; however, the former narrows genetic improvements down to mere cell surface engineering, while the latter is less compatible with the integration of phycoremediation with bioenergy production, as it constrains contact between microalgae and the surrounding aqueous solution. An alternative strategy is establishing sufficient physical containment, combined with post-treatment of bioremediated wastewater, so as to assure full elimination of transgenic microalgae. In any case, application of transgenic microalgae to HM bioremediation seems inevitable, given the legalization history of GMOs.

Needless to say, the sequencing of more genomes and performance of high-throughput algomics and metagenomics analyses will be crucial to establish an accurate map of the interplay between molecular mechanisms linking HM toxicity to constituted adaptive responses in microalgae, which will eventually lead to the identification and characterization of novel genetic engineering targets. Furthermore, the integration of wastewater treatment with bioenergy production, using hydrothermal liquefaction in a closed-loop system, may address several issues associated with the use of transgenic microalgae, while allowing successful scale-up of this promising technology. Finally, reliable utilization of transgenic microalgae-mediated bioremediation of HM-contaminated wastewater will require accurate life cycle assessment and associated technoeconomic cost analysis. Otherwise, the socioenvironmental feasibility of the process will likely be compromised.

## 6. Conclusions

This review highlighted current knowledge on the cellular and molecular mechanisms associated to microalgae-mediated adsorption, intake, accumulation, and transformation of heavy metals from the medium, and specifically focused on the most promising genetic and metabolic engineering targets, aimed at improving their bioremediation capacity via state-of-the-art molecular and bioinformatic tools. Despite being one of the most sophisticated biological systems regarding resistance to and transformation of heavy metals, the evidence reviewed and discussed shows, beyond doubt, that further improvements are possible in such phycoremediation ability—namely through specifically designed genetic modifications. Acceleration, yet under tight control, of the underlying forces of natural evolution will likely play an important role in our quest toward restoration of the lost ecological balance, while furthering knowledge of the processes supporting life. Genetic engineering holds an immense potential, yet careful experimentation and implementation are vital. Harnessing this potential and converting it into a useful technology for a better future demands, indeed, rational approaches, rather than prohibitive regulations *tout court* and strict banning; carefully designed bench- and industrial-scale efforts, complemented by open and transparent communication between science and technology stakeholders, and to the society at large, constitute the only reasonable and effective path thereto.

## Figures and Tables

**Table 1:** Genetic and metabolic engineering tools available for microalgal strain improvement.

Genetic and Metabolic Engineering Tools	Type	Specifics	Advantages and Disadvantages
CRISPR/Cas systems	Gene editing	A single guide RNA (sgRNA) drives the Cas protein to a matching DNA sequence on the host cell genome—which is degraded by Cas protein to create knockout and deletion mutants; and replaced by a new gene to generate insertion and knockdown mutants, with the aid of non-homologous, end-joining machinery	Allows manipulation of any DNA sequences; multiple mutations are possible; comparably more whole genome data are required; possibility of off-target events; large size of Cas makes cell delivery challenging
ZFNs	Gene editing	An array of site-specific DNA-binding domains that recognizes two sequences flanking a specific site, attached to the endonuclease domain of bacterial FokI restriction enzyme; upon binding, FokI domains dimerize and cleave DNA at the site, which will be repaired by the DNA repair machinery of the cell	Comparably high probability of off-target events; complicated programming is required; limited possible genomic target sites; only single mutations are possible
TALENs	Gene editing	Tandem arrays with 10 to 30 repeats that bind and recognize extended DNA sequences, attached to the endonuclease domain of bacterial FokI restriction enzyme; upon binding, FokI domains dimerize and cleave DNA at the site, which will be repaired by the DNA repair machinery of the cell	Comparably high probability of off-target events; complicated programming is required; limited possible genomic target sites. large TALENs hard to express or transfect into the cell; only single mutations are possible
Cre/*loxP* recombination systems	Gene editing	Two *loxP* sequences flanking the target gene interact with Cre recombinase for insertion of a new gene fragment or deletion of the one targeted	Simple and efficient; allows multiple genome integration; occasional off-target events are possible; toxicity of Cre for Cre-expressing cells
Modular cloning systems	Synthetic biology	Design and construction of expression vectors by providing a library of genetic building blocks (e.g., promoters, UTRs, terminators, tags, reporters, antibiotic resistance genes, introns)	Simple and efficient; high flexibility; expression of multiple transgenes possible; developed for limited range of species
RNAi technology	Gene silencing	Small RNA molecules bind to target mRNAs to form double-stranded RNAs, which are degraded by RNA-induced silencing complex (RISC)—and cause sequence-specific suppression of gene expression, through translational or transcriptional repression	Simple and efficient; occasional off-target effects; produces hypomorphic phenotypes, which do not always mirror the complete loss-of-function that often occurs with genetic mutation; nuclear transcripts—e.g., long non-coding RNAs or lncRNAs, more difficult to effectively target
Multiomics technologies	Omics	Analysis of whole-cell biochemical information of cell through genomics, transcriptomics, proteomics, metabolomics, interactomics, phenomics, meta-omics, etc.	Provide snapshot of response of cell or whole ecosystem to environmental changes; improves data comparability; time-consuming; high cost; sophisticated, expensive equipment required
Online databases	Bioinformatics	Integrated online platforms e.g., ChlamyCys, Greenhouse, Diatom EST database, Alga-PrAs, Cyan-Omics, and KEGG	Functional interpretation of genes and elucidation of their underlying biological themes via integrated annotation and expression data; freely available
Flux balance analysis	Systems	All relevant metabolic information of an organism (e.g., genes, enzymes, reactions) are collected, and analyzed with the aid of a mathematical model within the perspective of the entire network, and applied to make predictions and genome reconstruction	Allows tailor-made design of cell factories aimed at maximum efficiency; time-consuming; still in its infancy
Illumina microarray technology	Sequencing	Tiny silica microbeads are housed in carefully etched microwells, and coated with multiple copies of an oligonucleotide probe targeting a specific DNA or RNA sequence; upon excitation by laser, binding of each probe to a complementary sequence in sample results in signal that conveys information to the detector	Fast and robust; no prior knowledge of gene sequences required; potent means for gene sequence and expression analysis; high cost

**Table 2:** Genetic engineering targets anticipated to improve microalgal HM bioremediation capacity.

Approach	Targets
**Metal transportation**	NRAMP, ZRT, IRT, ZIP, FTR, CTR, CDF, HMA, FPN, Ccc1/VIT1, PTA, AQP, MTP, PMA, V-ATPase, V-PPase, MRP, ATM/HMT, PDR, YSL, CAX, MFS
**Metal chelation**	MTs, PCs, GSH, PPK, VTC, PPX, Pro (P5CS), His (HISN3), Cys (HAL2), Ser (SDC1), glycine-betaine, and organic acids
**Metal biotransformation**	ChrR, arsC, CrACR2s, MerA/B/P/C/T/F, SMT, CYPs
**Oxidative stress response regulation**	Trx, HSPs, carotenoids, SOD, POD, CAT, GPX, GST
**Metal stress response regulation**	MTF-1, C2H2, AP2, MYB, bHLH, YABBY, bZIP, SBP, HB, WRKY13, CRR1, ABI5, GATA, CKs (IPT and CKX1), GA, ABA, BRs, JA, ET, SA, miRNAs (miR398, miR319, miR390, miR393, miR171, miR395, miR397, miR408, and miR857)
**Cell-surface bioengineering**	MTs, PCs, 6x-His, CXXEE, MerR, GST, NikRm, ModE

## Data Availability

Not applicable.
